# Molecular imaging reveals biodistribution of P-cadherin LP-DART bispecific and trafficking of adoptively transferred T cells in mouse xenograft model

**DOI:** 10.18632/oncotarget.27544

**Published:** 2020-04-14

**Authors:** Vijay R. Gupta, Adam Root, Timothy Fisher, Rand Norberg, John David, Tracey Clark, Justin Cohen, Chad May, Anand Giddabasappa

**Affiliations:** ^1^Global Science & Technology (GST) – Comparative Medicine, Pfizer Global Research Development and Medical, San Diego, CA 92121, USA; ^2^BioMedicine Design, Cambridge, MA 02139, USA; ^3^Oncology Research and Development, San Diego, CA 92121, USA; ^4^PDM Biotherapeutics, Pfizer Inc., San Diego, CA 92121, USA

**Keywords:** bispecific antibody, tumor targeting, biodistribution, T cells, molecular imaging

## Abstract

P-cadherin-LP-DART is a bispecific antibody targeting P-cadherin expressed on the tumor cells and CD3 on the T-cells. Previously we demonstrated the development and efficacy of P-cadherin-LP-DART in *in vitro* and *in vivo* models. Here, we evaluated the three pillars: exposure, targeting specificity and pharmacodynamic modulation for P-cadherin-LP-DART using fluorescence molecular tomography (FMT). Bispecific antibodies and T-cells were conjugated with a near-infrared fluorophores: VivoTag^®^680XL (VT680) and CellVue^®^NIR815 (CV815), respectively. *In vitro* binding and cytotoxic T-lymphocyte assay demonstrated that P-cadherin-LP-DART significantly retained its properties after VT680 conjugation. *In vivo* FMT imaging was performed to determine the bispecific biodistribution and T-cell trafficking in HCT-116 xenograft model. Peak tumor exposure (2.71%ID) was observed at 96 hr post-injection with measurable quantity even at 240 hr (1.46%ID) (Pillar 1). P-cadherin-LP-DART accumulation in tumor was 20-25 fold higher compared to Control-LP-DART demonstrating the targeting specificity (Pillar 2). Imaging after engraftment of CV815 labeled T-cells showed P-cadherin-LP-DART mediated T-cell trafficking in tumors (Pillar 3). This study harnessed the multichannel capability of FMT and demonstrated the targeting of drug and trafficking of T cells to tumors, simultaneously. Our results show the impact of molecular imaging in demonstrating three pillars of pharmacology, longitudinally and non-invasively.

## INTRODUCTION

Antibody-based therapeutic platforms have revolutionized the biopharmaceutical landscape in last two decades [1]. Advances in protein engineering have transformed the antibody-based therapeutics to overcome limitations including antigen targeting, immunogenicity, pharmacokinetics and manufacturability [1, 2]. Bispecific antibodies are one of these innovative platforms that can co-engage two different antigens at the same time [2, 3]. Renewed interest in cancer immunotherapy has focused on bispecific antibodies as a preferred therapeutic modality used to direct immune effector cells to tumors expressing a target antigen to trigger an anti-tumor cytotoxic response [2, 4]. Immune effector cells that can be engaged by bispecific antibodies include T cells, Natural Killer (NK) cells and Macrophages. Effector T cells have the ability to expand rapidly upon activation and also have the potential to generate immunological memory, thereby generating robust and durable anticancer response [5]. Mechanistically, bispecific antibodies targeting effector T cells co-engage the CD3 epsilon subunit on the T cells and specific antigen (s) on tumor cells to form a bispecific mediated synapse. Target co-engagement by bispecific antibodies leads to activation of the T cell receptor (TCR) signaling cascade resulting in release of granzymes and perforin molecules that results in tumor cytotoxicity. Currently, two T cell targeting bispecific antibodies have been approved to treat cancer and an additional 50 molecules are in different stages of clinical development [3, 6–8].

**Figure 1:**
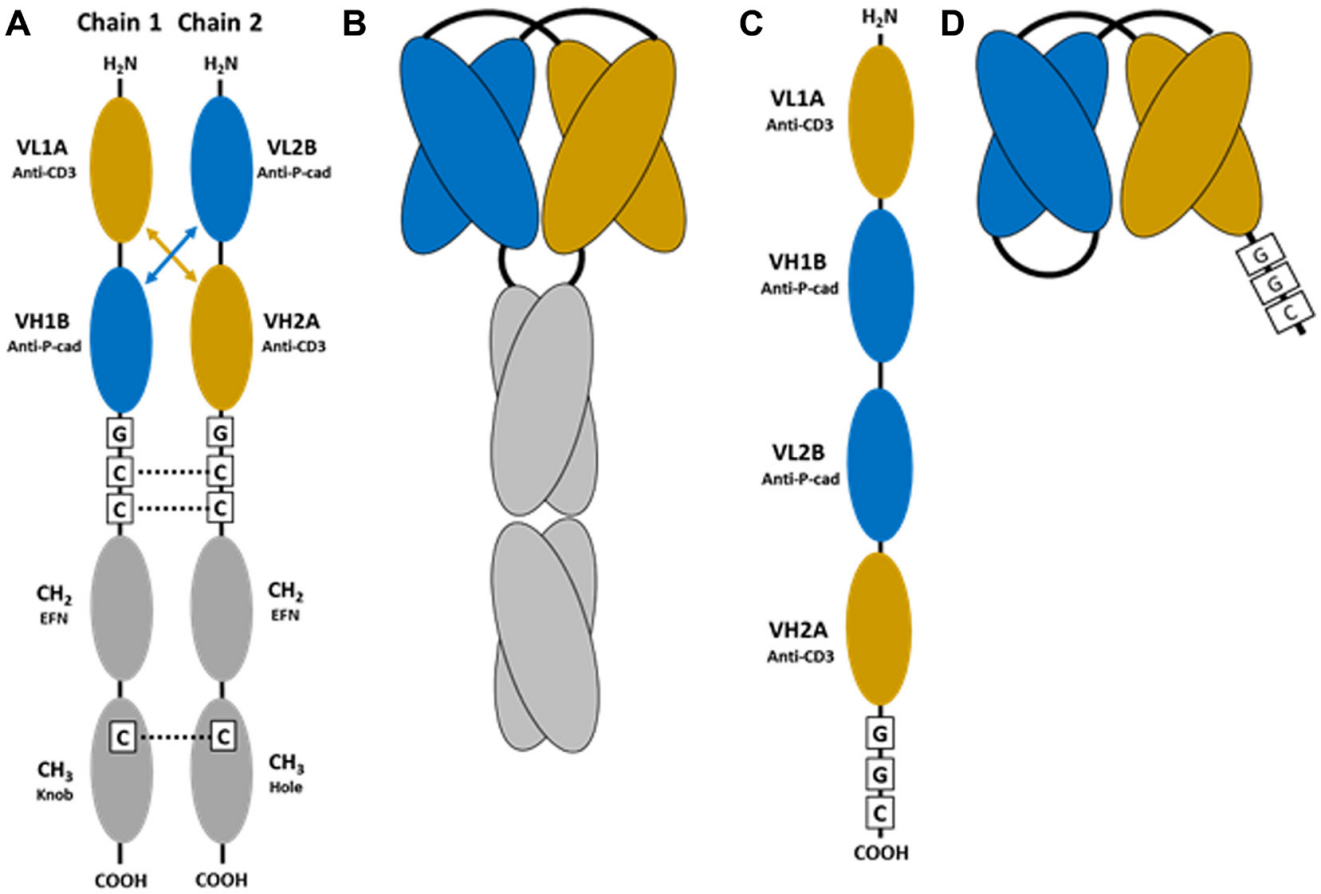
Schematic of the bispecific reagents used in this study. (**A**) Representation of the individual chains of anti-P-cadherin human IgG1 Fc-containing DART, also referred to as P-cadherin LP-DART. Cysteine residues introduced for interchain disulfide formation are indicated by C. EFN, effector function null mutations in CH2. Knob, hole: complementary mutations introduced to force Fc heterodimerization [10–12]. (**B**) Schematic representation of the folded P-cadherin LP-DART protein after heterodimerization. (**C**) Representation of the anti-P-cadherin single chain diabody (scDb) DART linear expression construct. Cysteine residue introduced for site specific conjugation at the C-terminus is indicated by C. (**D**) Schematic representation of the folded anti-P-cadherin DART (scDb) protein.

Bispecific antibodies with affinity to multiple targets offer greater functionality when compared to traditional monoclonal antibodies. However, development of these next generation biologics is increasingly complex and often requires intricate protein engineering approaches to create novel scaffolds and technology platforms. The bispecific antibody formats can be broadly categorized as a) conventional IgG like bispecific antibodies that retain Fc-mediated effector antibody-dependent cellular cytotoxicity (ADCC) and complement-dependent cytotoxicity (CDC) functionalities or b) novel non-IgG like bispecific antibodies that generally have dual binding functionality with minimal or no effector functions [4, 7]. The dual-affinity re-targeting (DART) platform is a bispecific molecular format that can co-engage the TCR/CD3 complex on effector T cells and a targeting antigen on tumor cells [9]. The DART format is a bispecific diabody comprised of two distinct antigen-specific single chain variable fragments (scFv) joined on either two separate chains or as a single chain. The LP-DART format fuses this bispecific diabody to an engineered human IgG1 Fc domain that is devoid of Fc mediated effector functions (Figure 1A–1D) [10–12].

P-cadherin is a classical cadherin that is part of the adherens junction and mediates calcium dependent cell-cell adhesion. Differential upregulation of P-cadherin is observed in a certain solid cancer types including breast, gastric, endometrial, colorectal and pancreatic cancers when compared to normal tissues [13, 14]. P-cadherin upregulation has also been shown to correlate with poor survival of breast cancer patients [15]. Thus P-cadherin is an attractive antigen to target tumors with elevated expression of this protein. P-cadherin LP-DART is a bispecfic molecule that uses the DART platform to bind CD3 epsilon on the T cells and P-cadherin on the tumor cells. The human Fc region of P-cadherin LP-DART molecule is engineered to eliminate Fc gamma receptor binding but retain neonatal Fc receptor (FcRn) binding and thus offer extended plasma half-life [10, 16]. P-cadherin LP-DART has demonstrated potent cytotoxic T-cell mediated tumor cell killing in *in vitro* and *in vivo* models [16]. To further expand our understanding of the pharmacology of P-cadherin LP-DART (i.e., the exposure at the target site and the recruitment of T cells in tumors), we have utilized fluorescence molecular tomography (FMT) imaging [17, 18]. Longitudinal *in vivo* biodistribution, tissue exposure and tumor targeting of a P-cadherin LP-DART was assessed using FMT after labeling with near-infra red (NIR) fluorophore VivoTag^®^680XL (here after referred as VT680). Additionally, we explored the possibility of adopting FMT imaging to provide mechanistic insights by visualizing the T cell redistribution and tumor trafficking dynamics upon treatment with P-cadherin LP-DART.

## RESULTS

### Evaluation of binding and functional properties of VT680 conjugated P-cadherin LP-DART

**Figure 2:**
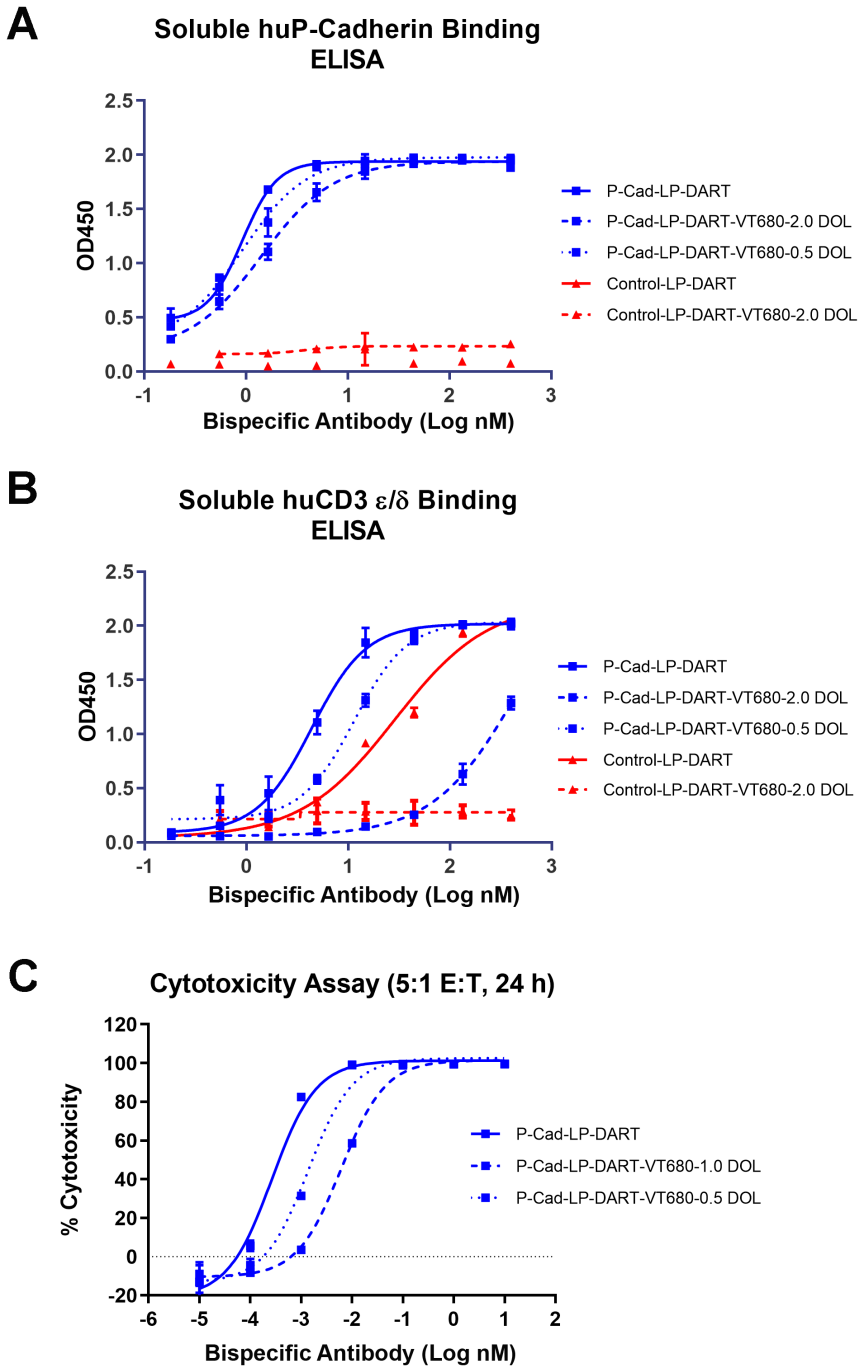
*In vitro* assays to evaluate the effect of labeling of VivoTag^®^680XL to P-cadherin LP-DART. ELISA based assay was used to evaluate the effect of VT680 labeling on binding of P-cadherin LP-DART to human P-cadherin and human CD3. The proteins were coated on to the plates and incubated with serial dilutions of P-cadherin LP-DART-VT680 or Control LP-DART-VT680 for 1 hr at 37°C. The bound P-cadherin LP-DART was quantified using IgG-HRP conjugate followed by calorimetric quantitation. (**A**) The labeling of VT680 to P-cadherin LP-DART had a DOL dependent effect on binding to human P-cadherin and Control LP-DART had no binding to human P-cadherin. (**B**) The labeling of VT680 to P-cadherin LP-DART and Control LP-DART affected binding to human CD3. P-cadherin LP-DART retained moderate binding even at a DOL of 2.0, whereas Control LP-DART lost its binding to human CD3 protein. (**C**) CTL Assay: Firefly luciferase expressing HCT116 cells and expanded human CD3+ T lymphocytes were co-incubated with increasing concentrations of P-cadherin LP-DART with different DOL of VT680. After 24 hr the remaining viable cells were quantified by measuring the luciferase activity. The relative cytotoxicity observed at various concentrations of P-cadherin LP-DART-VT680 was plotted against the PBS treated samples. VT680 conjugation decreased the cytotoxic ability in a DOL dependent manner.

The bispecific antibodies were labeled with amine-reactive fluorophore VT680 using NHS (N-hydroxysuccimide) chemistry. *In vitro* P-cadherin and CD3 binding property and functional cytotoxic activity of the VT680 bispecific antibody conjugates were evaluated prior to the *in vivo* studies. P-cadherin LP-DART binds specifically to human P-cadherin and CD3ε proteins, whereas the negative control (Control LP-DART) binds only to the human CD3ε protein. Fluorophore labeled P-cadherin LP-DART and Control LP-DART were compared with respective unlabeled counterparts for binding to soluble human P-cadherin and soluble human CD3 epsilon/delta (hCD3 ε/δ) (Figure 2A and 2B). Dose-dependent binding curves demonstrate that VT680 labeling of P-cadherin LP-DART minimally affect binding to P-cadherin when compared to unlabeled antibody. The half maximal effective concentration (EC50) for P-cadherin binding was 0.92 nM, 0.93 nM and 1.42 nM for unlabeled P-cadherin LP-DART, and for P-cadherin LP-DART-VT680 with degree of labeling (DOL) of 0.5 and 2.0, respectively. However, the VT680 labeling reduced the binding of P-cadherin LP-DART to soluble hCD3 ε/δ with EC50 values of 4.34 nM, 11.47 nM and > 100 nM for unlabeled antibody, DOL of 0.5 and 2.0, respectively. This data suggested that the CD3 binding domain was more sensitive to VT680 labeling than the P-cadherin binding domain.

*In vitro* functional activity of the P-cadherin LP-DART and P-cadherin DART was evaluated using the cytotoxic T lymphocyte (CTL) assay. The EC50 values were 0.3 pM, 1.4 pM and 6.2 pM for the unlabeled P-cadherin LP-DART, DOL 0.5 and 1.0, respectively (Figure 2C). P-cadherin LP-DART-VT680 with DOL 2.0 still retained cytotoxic activity, whereas the unlabeled control-LP-DART of control-LP-DART-VT680 at DOL 2.0 did not show any cytotoxicity (Supplementary Figure 1). Similarly, the P-cadherin DART as single chain diabody (scDb) showed a reduction in cytotoxic activity after conjugation to VT680 at DOL of 1 (Supplementary Figure 2A). These results suggested that VT680 labeling had a DOL dependent effect on cytotoxicity, but still retained potent (low pM) cell killing potential at DOL of 1.0.

### *In vivo* biodistribution and tumor targeting kinetics of P-cadherin LP-DART in HCT-116 xenograft model

**Figure 3:**
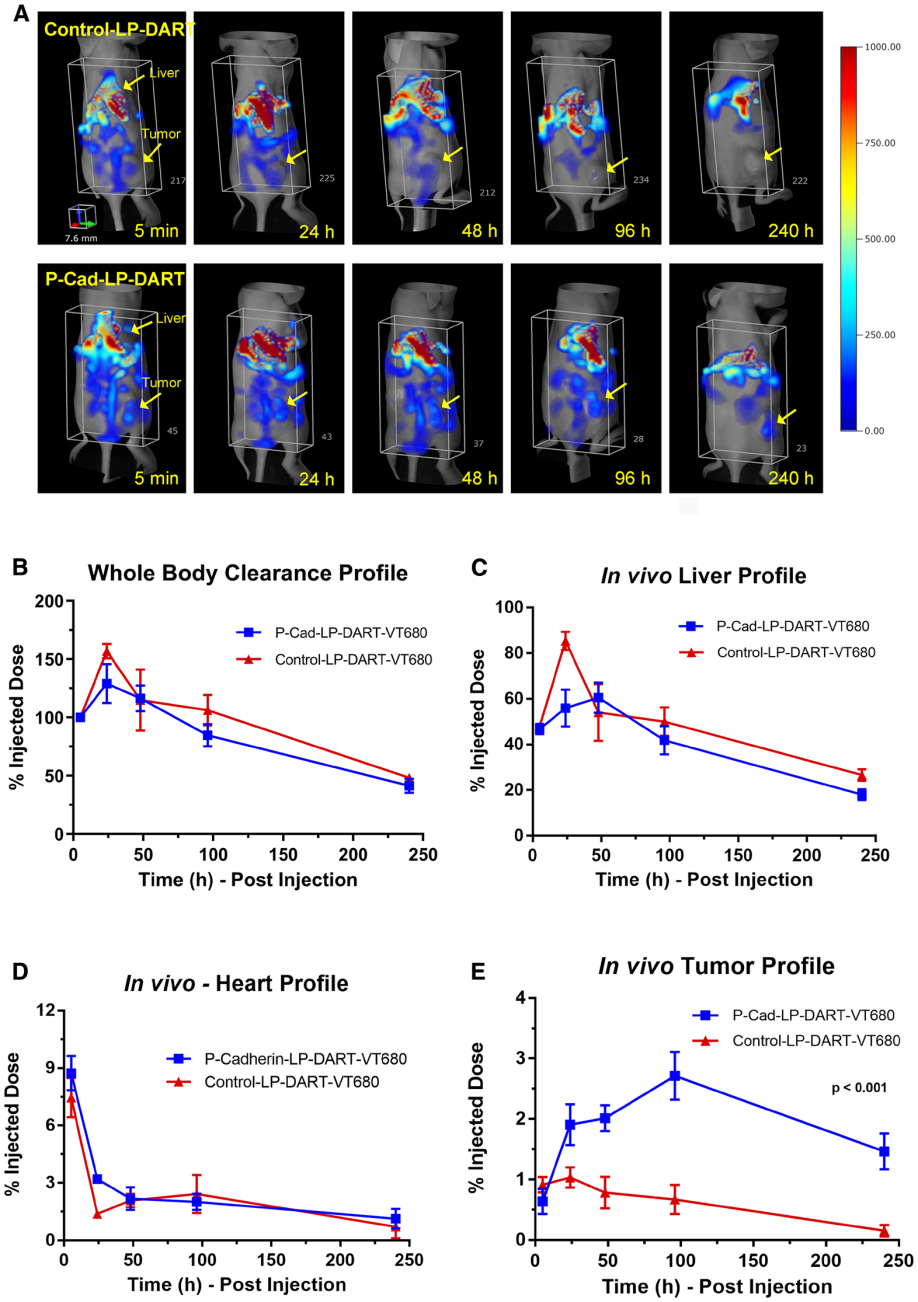
*In vivo* biodistribution and tumor targeting profile of P-cadherin LP-DART-VT680 and Control LP-DART-VT680. Female nu/nu mice bearing HCT116 xenografts were injected intravenously with P-cadherin LP-DART-VT680 and Control LP-DART-VT680 at doses equivalent to 1 nmol of VT680 (~1.4 mg/kg). FMT imaging was performed using the 680 laser at 5 min, 24, 48, 96 and 240 hr post-injection. (**A**) Representative 3-dimentional FMT images showing the whole-body (torso) biodistribution of P-cadherin LP-DART-VT680 and Control LP-DART-VT680. Arrows indicate the location of tumor and liver. (**B**) Relative whole-body profile of P-cadherin LP-DART-VT680 and Control LP-DART-VT680. (**C**) Relative liver profile of P-cadherin LP-DART-VT680 and Control LP-DART-VT680. (**D**) Upper torso was imaged separately to image heart and brain region. Relative heart profile of P-cadherin LP-DART-VT680 and Control LP-DART-VT680 shows a biphasic clearance profile. (**E**) Relative tumor accumulation of P-cadherin LP-DART-VT680 and Control LP-DART-VT680. No major difference was observed between P-cadherin LP-DART-VT680 and Control LP-DART-VT680 in the profiles of whole-body, liver, and heart. Tumor profile showed a significantly increased accumulation of P-cadherin LP-DART-VT680 compared to Control LP-DART-VT680. *n* = 4–6/group; ± SEM is represented in the graphs; Tumor accumulation: *p* value < 0.001, 2-way ANOVA.

We have previously shown the differential expression of P-cadherin in variety of human tumor cell lines [10]. The biodistribution and tumor targeting kinetics of P-cadherin LP-DART was evaluated in high P-cadherin expressing HCT116 xenograft model by longitudinal FMT imaging. Representative three-dimensional (3D) whole-body (torso) images showing the time course for both P-cadherin LP-DART and Control LP-DART are represented in Figure 3A. A 3D region of interest (ROI) was placed in the torso area to represent signal from the whole-body. A minimum background signal (< 5%) was observed from the cranial region and thus was not included in quantitation. Quantitation of fluorescence by FMT in whole-body (Figure 3B) and liver (Figure 3C) showed initial spike in signal at 24 hr time point followed by gradual decrease in signal due to sustained clearance of the probes. A 3D ROI was placed around the heart region to quantify the heart. Since we did not anticipate any binding to the heart tissue we considered this fluorescence signal as a measurement for blood. Overall, the whole-body, heart and liver profiles were similar in both P-cadherin LP-DART and Control LP-DART groups (Figure 3B–3D). However, we observed a selective tumor accumulation of P-cadherin LP-DART that gradually increased with time and peaked at 96 hr post-injection (2.71% Injected Dose (ID)) when compared to Control LP-DART which showed significantly lower accumulation (0.67%ID) (Figure 3E). Although the tumor accumulation in both the groups declined after 96 hr, there was significant VT680 signal in the P-cadherin LP-DART group at 240 hr post-injection (1.46%ID) compared to Control LP-DART (0.16%ID), suggesting a prolonged retention of the drug in the tumor. Overall, *in vivo* FMT imaging data showed selective tumor accumulation of P-cadherin LP-DART when compared to the non-specific Control LP-DART.

To compare the tumor targeting and clearance profiles of P-cadherin LP-DART and its scDb without Fc component, we evaluated whole-body biodistribution of P-cadherin DART-VT680 in HCT116 xenograft model (Supplementary Figure 2B and 2C). To our surprise, the whole-body profile of P-cadherin DART was different than the P-cadherin LP-DART. Significant accumulation of P-cadherin DART was observed in the kidneys as early as 6 hr post-injection (Supplementary Figure 2C). The kidney signal was prominent compared to liver or the tumor suggesting that the P-cadherin DART is being cleared rapidly by the kidneys. Though moderate tumor accumulation (~1.5%ID, *in vivo*) was observed at 48 hr post-injection, as expected P-cadherin DART cleared rapidly by 96 hr from the tumor (< 0.5%ID, *in vivo*) and the whole body (Supplementary Figure 2B and 2C).

### *Ex vivo* quantification of P-cadherin LP-DART in tumor and other organs

**Figure 4:**
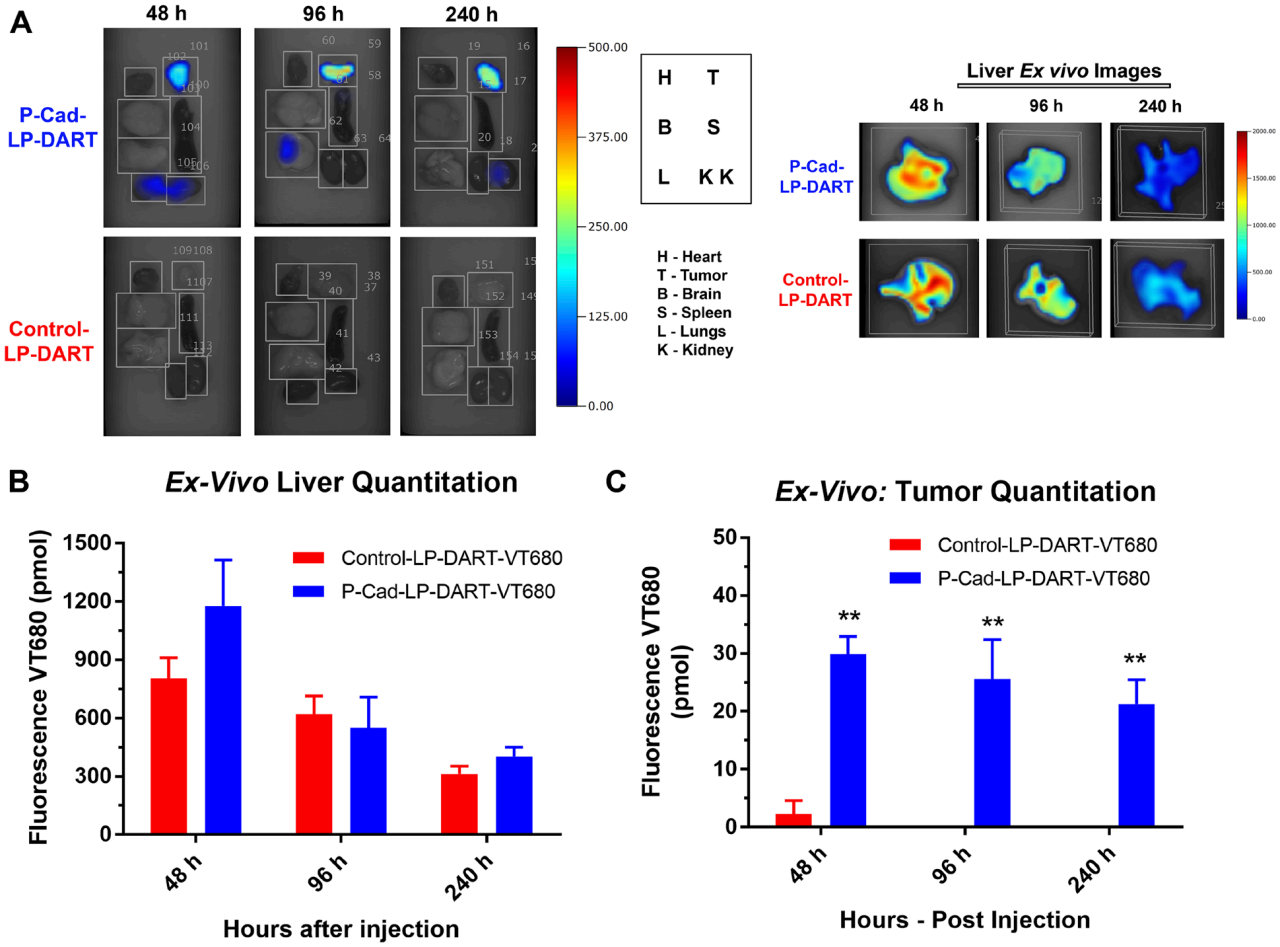
*Ex vivo* imaging and quantitation of accumulation in tumor and other organs. (**A**) *Ex vivo* FMT imaging panel showing the accumulation of P-cadherin LP-DART-VT680 and Control LP-DART-VT680 at 48, 96 and 240 hr in various organs: Tumor (T), Heart (H), Brain (B), Spleen (S), Lungs (L), Kidneys (K). No notable signal observed from any organs in this image except for tumor. Liver was imaged as a separate panel due to its size and was quantified. (**B**) *Ex vivo* quantitation of liver: No significant differences in liver was observed between P-cadherin LP-DART-VT680 and Control LP-DART-VT680 at 48, 96 and 240 hr. (**C**) *Ex vivo* quantitation of tumor showed significantly increased accumulation of P-cadherin LP-DART-VT680 compared to Control LP-DART-VT680 at 48, 96 and 240 hr. *n* = 3–4/group; ± SEM is represented in the graphs; ^**^ = *p* value < 0.01.

FMT quantification of whole-body represents signal from accumulation of labeled reagent (probe) in tumor and other tissues in the torso region, as well as signal due to the probe in the circulation. To eliminate the residual signal contributed from the probe in circulation/vasculature, we perfused the whole-body with saline and quantified the tumor and selected tissues (brain, heart, lungs, kidneys, liver and spleen) *ex vivo* by FMT at 48, 96 and 240 hr (Figure 4A–4C and Table 1). Liver was the main clearance site for both the P-cadherin LP-DART and Control LP-DART (Figure 4A and 4B). The kidneys and lungs also showed minor accumulation (Figure 4A), albeit at very low levels compared to liver. There was no considerable signal observed in other organs that were quantitated *ex vivo*. The comparison of quantitative values suggested that both P-cadherin LP-DART and Control LP-DART accumulated at similar levels in all the organs, except tumor. Quantitation of *ex vivo* FMT signal in tumor showed at least a 15-fold higher accumulation of P-cadherin LP-DART compared to Control LP-DART (29.9 pmol vs 2.31 pmol at 48 hr post-injection) (Figure 4C and Table 1). Although there was a trend towards decrease in tumor quantities between the 48, 96 and 240 hr time-points (29.9, 25.64 and 21.28 pmol) in the P-cadherin LP-DART group, they were not statistically different. No detectable signal was observed in the *ex vivo* tumors of Control LP-DART at 96 and 240 hr time-points (Figure 4C). Overall *ex vivo* FMT imaging confirmed the specific accumulation of P-cadherin LP-DART in the tumors as observed by *in vivo* imaging. FMT data also showed that liver was acting as a sink and the major site for clearance of both P-cadherin LP-DART and Control LP-DART (Table 1).

**Table 1:** *Ex vivo* FMT quantitation of P-cadherin LP-DART and control LP-DART accumulation in tumor and selected organs

P-cadherin LP-DART							
Time	Tumor	Liver	Brain	Heart	Kidneys	Lungs	Spleen
**48 h**	29.90^**^	1177.12	0.29	0.00	9.51	11.13	0.47^**^
**96 h**	25.61^*^	549.29	0.63	0.87	18.26	9.49	0.66^**^
**240 h**	21.29^**^	402.52	0.04	0.24	5.86	0.01	0.09

Control LP-DART							
Time	Tumor	Liver	Brain	Heart	Kidneys	Lungs	Spleen
**48 h**	2.31	805.33	0.08	0.06	6.46	1.48	0.05
**96 h**	0.00	619.42	0.07	0.33	30.93	0.02	0.07
**240 h**	0.00	313.05	0.00	0.00	4.82	0.00	0.00

Significantly higher accumulation of P-cadherin LP-DART was observed in tumor, compared to control LP-DART at all three *ex vivo* time points, 48 h, 96 h and 240 h. No significant difference between P-cadherin LP-DART and control LP-DART was observed in liver, brian, heart, kidneys and lungs. Although spleen showed significantly higher accumulation of P-cadherin LP-DART compared to control LP-DART at 48 h and 96 h, the quantities were very low.

### Mechanistic characterization of P-cadherin LP-DART induced *in vivo* T cell trafficking to tumors in HCT-116 xenograft model

**Figure 5:**
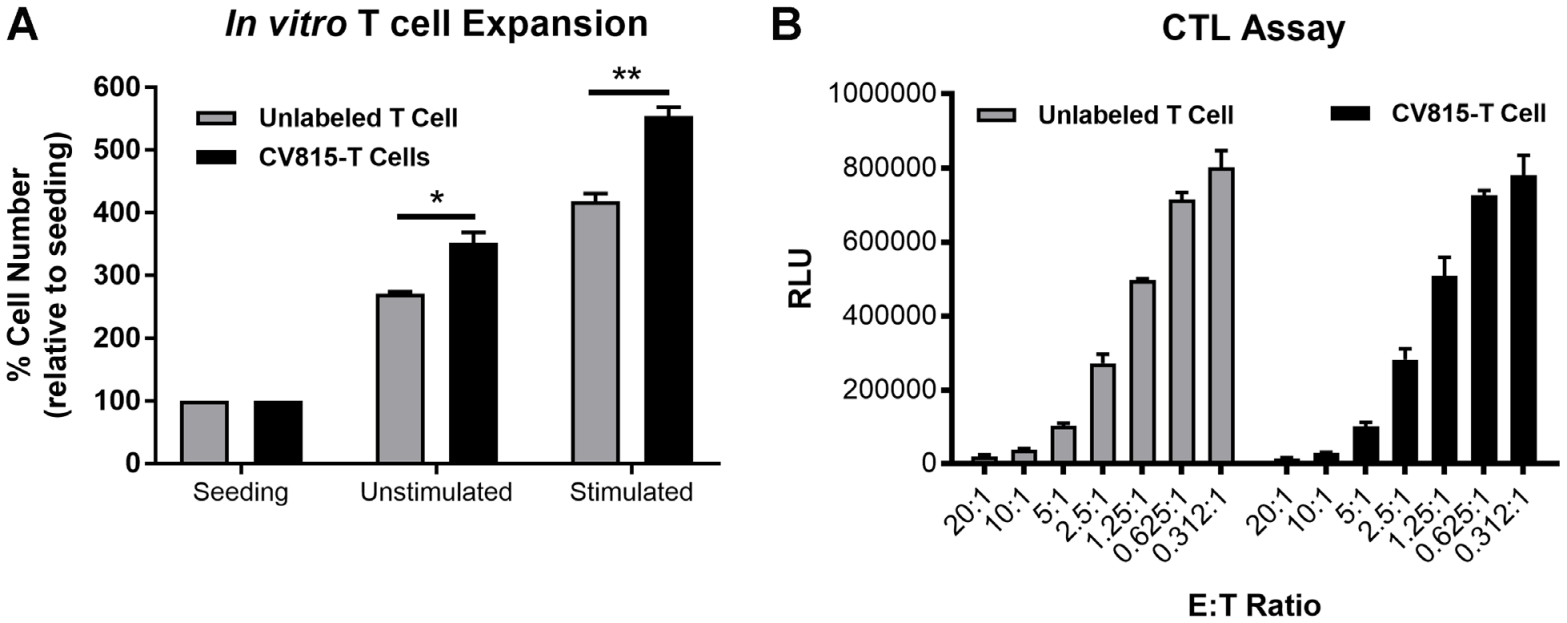
Effect of *in vitro* labeling of CD3+ T cells with CellVue^®^ NIR815 fluorophore. (**A**). *In vitro* T Cell expansion of CV815 labeled CD3+ T cells. Unlabeled and CV815-T cells were incubated under unstimulated or CD3/CD28 stimulation condition for 48 hr and quantified the number of cells using CellTiter-Glo^®^ Luminescent Cell Viability kit. (**B**) *In vitro* CTL assay was performed by mixing different E: T ratios with 20 pM P-cadherin LP-DART for 24 hr. The number of viable target cells was estimated using ONE-Glo^®^ luciferase kit. ± SEM is represented in the graphs; ^*^ = *p* value < 0.05; ^**^ = *p* value < 0.01.

P-cadherin LP-DART is a CD3 bispecific antibody that can mechanistically recruit effector T cells to tumors and trigger anti-tumor cytotoxic response. Previously, we have shown that P-cadherin LP-DART can regress HCT116 tumor xenografts [16]. Here we used FMT imaging to study T cell trafficking to tumors in HCT116 xenograft model, upon treatment with P-cadherin LP-DART. T cells were labeled with a membrane labeling NIR fluorophore CellVue^®^ NIR815 (here after referred as CV815). To assess if CV815 labeling of T cells affected its survival or function, we performed *in vitro* T cell expansion and cytotoxicity assays. CV815-T cells and unlabeled T cells were stimulated with CD3/CD28 for 48 hr and assessed for expansion. Unstimulated cells in the media increased by ~2-3 fold, whereas the stimulated T cells were increased by 4–5 fold, relative to number of cells seeded (Figure 5A). The CV815 labeled T cell expansion was greater than unlabeled T cells. The cytotoxic activity of CV815 labeled T cells and unlabeled T cells was evaluated by incubating with luciferase expressing HCT116 cells at different effector: target cell (E: T) ratios (20:1 to 0.312:1). Both CV815 labeled and unlabeled T cells showed similar cytotoxic potential at various E: T ratios tested (Figure 5B). These results suggested that CV815 labeling did not affect the T cell function and hence were optimal for use in *in vivo* FMT imaging.

**Figure 6:**
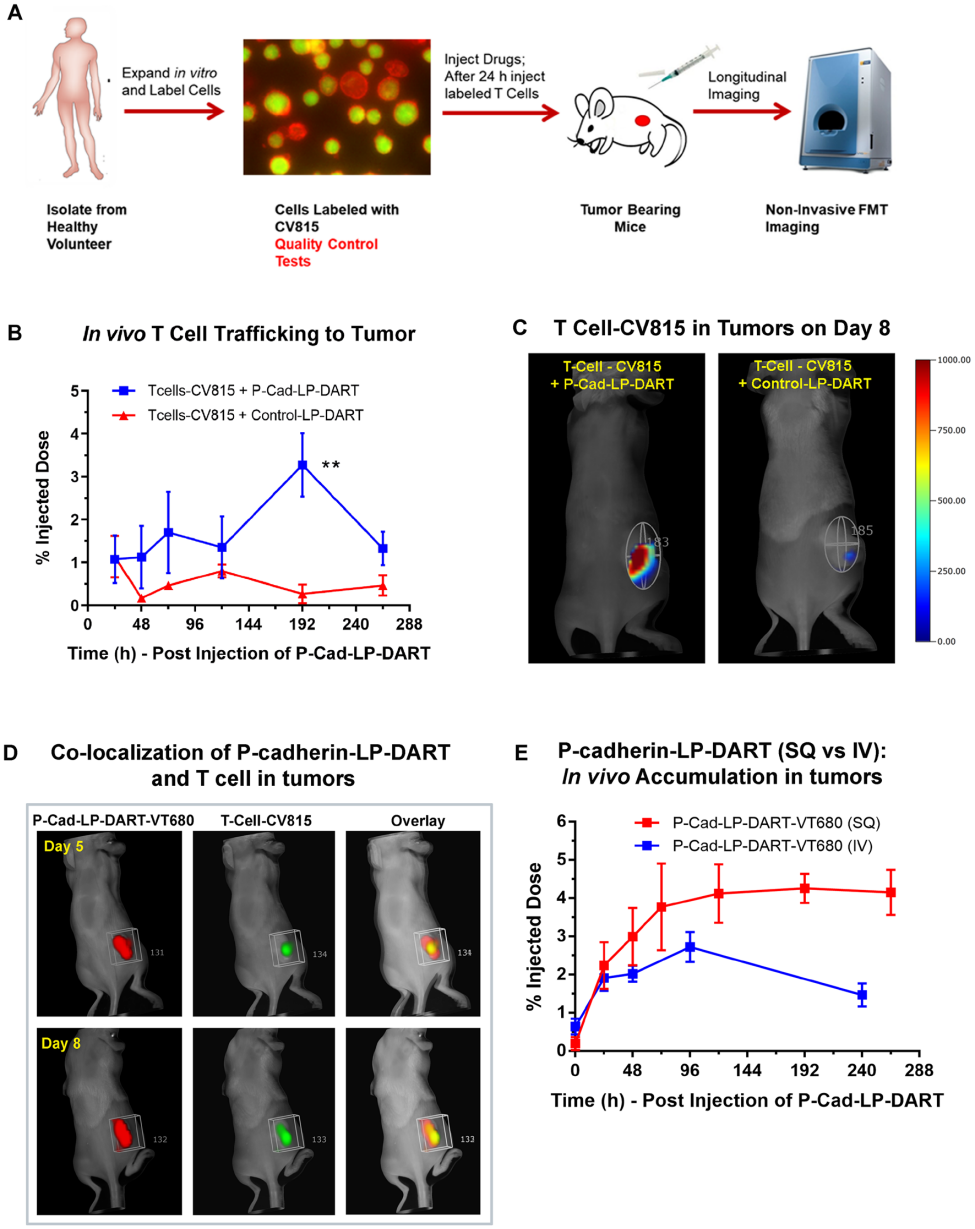
*In vivo* T cell trafficking in HCT-116 xenograft model by FMT imaging. Female NSG mice bearing HCT116 xenografts were injected subcutaneously with P-cadherin LP-DART, P-cadherin LP-DART-VT680 or Control LP-DART at 2 mg/kg dose. After 24 hr the CV815-T cells (5 × 10^6^ cells) were injected intravenously. FMT imaging was performed longitudinally using the 680 nm laser for VT680 and the 800 nm laser for CV815. (**A**) Schematic showcasing the design of T cell trafficking study. (**B**) Profile of *in vivo* tumor trafficking of CV815-T-Cells in groups treated with unlabeled P-cadherin LP-DART and Control LP-DART. No significant difference of CV815-T-Cells was observed in until 120 hr post-injection. At 192 hr there was a significant increase in CV815 signal in tumors (*n* = 3–4/group; ± SEM is represented at all time points; *t*-test, ^**^ *p*-value < 0.01). (**C**) FMT images on day 8 (192 hr) showing the CV815-T cells in tumor of mice treated with unlabeled P-cadherin LP-DART whereas minimal signal was observed in Control LP-DART treated mice. (**D**) Representative FMT images on day 5 and 8 from a separate cohort of animals showing co-localization of P-cadherin LP-DART-VT680 (red) with CV815-T cells (green). (**E**) Comparison of profiles of tumor accumulation when P-cadherin LP-DART-VT680 was administered via intravenous route (IV) and subcutaneous route (SQ). FMT profile shows a slower, sustained and a slightly increased P-cadherin LP-DART-VT680 accumulation by subcutaneous administration.

To determine the biodistribution of CV815-T cells *in vivo* and to select the concentration for T cell trafficking study, we performed a pilot study in naïve NSG mice. Different concentrations (2 – 10 × 10^6^ cells) of stimulated CV815-labeled T cells were injected and imaged 24 h post-engraftment (Supplementary Figure 3). The T cells were primarily localized in spleen and liver in a concentration dependent fashion. We determined that the mice injected with 5 × 10^6^ cells provided signal above background and was not saturated in the major organs, spleen and liver. To assess P-cadherin LP-DART mediated T cell trafficking to tumors we adoptively transferred 5 × 10^6^ CV815-T cells to HCT116 xenograft bearing mice. Mice with tumors (~300 mm^3^) were randomized to treatment groups. Each group of animals received either unlabeled Control LP-DART; unlabeled P-cadherin LP-DART subcutaneously (SQ) on day 0. After 24 hr (day 1), CV815-T cells were injected intravenously (IV) and imaged by FMT longitudinally up to day 10 (Figure 6A). FMT imaging was performed 5 min after CV815-T-cell injection and the whole-body fluorescence signal was normalized as 100% injected dose. There was a decrease in tumor CV815 signal on all days compared to day 1 (~1.1%ID) in the Control LP-DART treated group (Figure 6B). The tumor CV815 signal in P-cadherin LP-DART treated group stayed relatively similar up to day 5 compared to day 1 (~1.1%ID). On day 8 there was an increase CV815 signal in tumors (~3.25%ID) suggesting increased infiltration T cells (Figure 6B). There was a 12-13 fold increase in CV815 signal in P-cadherin LP-DART group relative to the Control LP-DART group (3.25 vs 0.27%ID) on day 8 (Figure 6B) illustrating bispecific antibody mediated T cell trafficking to tumors (Figure 6C). On day 10 there was a significant decrease in tumor CV815-T cell signal which may be due to dilution of fluorescence due to T cell proliferation.

FMT4000 can image multiple near-infrared fluorescence channels without requiring spectral unmixing, thus we tried to visualize tumor specific co-localization of the P-cadherin LP-DART-VT680 and CV815-T cells. When the tumors were ~300mm3 P-cadherin LP-DART-VT680 was injected via subcutaneous route and 24 h later the CV815-T cells were injected via intravenous route. Figure 6D shows the pseudo-colored tumor ROI for P-cadherin LP-DART-VT680 (red) and CV815-T cells (green) and its overlay (yellow) on day 5 and day 8. There was no difference in the P-cadherin LP-DART-VT680 signal between day 5 and day 8, but an increase in CV815 signal in the tumors was visualized. Additionally, since we administered P-cadherin LP-DART-VT680 subcutaneously in this experiment we compared the tumor targeting profile with that of intravenous administration (Figure 3E) in the biodistribution study. An increased and sustained tumor targeting of P-cadherin LP-DART was observed when administered subcutaneously (peak on day 8 with ~4.25%ID) compared to the intravenous route (peak on day 4 with ~2.71%ID) (Figure 6E). This suggests that subcutaneous injection may act as a depot and may provide a potential advantage (Figure 6E).

## DISCUSSION

Molecular imaging like FMT and positron emission tomography (PET) are excellent non-invasive tools to evaluate the three pillars in drug discovery and development: exposure at the target site (Pillar 1), binding to the target (Pillar 2) and pharmacodynamic modulation (Pillar 3) [19, 20]. Imaging endpoints have important translational relevance as many of the endpoints can be adopted in clinical evaluation [20]. Here we have utilized FMT imaging to demonstrate the biodistribution and tumor targeting of P-cadherin LP-DART; the pharmacodynamic effect, i.e., the trafficking of T cells to the tumor microenvironment; the exposure profile of P-cadherin LP-DART, its scDb form (with no Fc domain) and the differences between administering P-cadherin LP-DART by subcutaneous vs intravenous route. Additionally, we harnessed the multi-channel capability of FMT imaging and examined the dynamics of targeting P-cadherin LP-DART and T cell to the tumors.

Innovation in protein engineering has resulted in a variety of bispecific formats for cancer immuno-therapy. Among these different bispecific platforms are the two drugs tested in patients: Catumaxomab and Blinatumomab [2, 7]. Catumaxomab is a 150 kDa hybrid IgG2 like molecule targeting epithelial cell adhesion molecule (EpCAM), a tumor antigen and CD3 on the T cells in contrast to Blinatumomab, which is a 55 kDa bispecific T cell engager (BiTE) targeting CD19 on the cancer cell and CD3 on the T cell. The pharmacokinetics of BiTE class of therapeutic is very interesting and is a differentiating property. T cell bispecifics possessing a full-length IgG Fc region demonstrate a longer half-life (2.13 days) as was observed in Catumaxomab. The incorporation of an Fc fragment increases the molecular weight and the circulation half-life through its interactions with the neonatal Fc receptor (FcRn) [21]. Blinatumomab, which lacks an Fc region and has only the single chain variable fragments (scFv) targeting CD19 and CD3 demonstrated a half-life of 2 hr [2, 7]. In our mouse studies using imaging-based approach we showed accelerated clearance to the P-cadherin DART (scDb) compared to the P-cadherin LP-DART, which has an engineered Fc region in the molecule. The half-life of P-cadherin LP-DART in a human FcRn transgenic mouse model was determined to be ~4.4 days [10]. Similar to our observation by FMT, PET imaging of ^89^Zr-AMG211, a BiTE molecule targeting carcinoembryonic antigen (CEA) and CD3e showed very rapid clearance through kidneys [22]. In this study the plasma half-life of ^89^Zr-AMG211 was determined to be ~1 hr. Such short plasma half-life requires continuous infusion and frequent administration of the drug, compared to weekly or bi-weekly administration of Fc-containing bispecific molecules [23, 24]. We also compared the exposure of P-cadherin LP-DART when administered intravenously or subcutaneously using FMT imaging. We saw an extended half-life with increased exposure of P-cadherin LP-DART at the tumor site by subcutaneous administration compared to intravenous route (4.14%ID on day 11 vs 1.46%ID on day 10). Since we are quantifying the fluorophore signal as a measure of the drug, it would be interesting to know the contribution of the intact P-cadherin LP-DART to its catabolized antibody.

Previously we demonstrated that the pharmacology of P-cadherin LP-DART is dependent on the expression of P-cadherin on the tumor cells. This study was performed using the HCT116 cell line which has been shown to express high levels of P-cadherin (antigen-binding capacity of 114,000) [10]. Targeting of P-cadherin LP-DART was dependent on the antigen specificity as the Control LP-DART which has similar affinity to T cells but does not bind to P-cadherin or the HCT116 cells, did not show any meaningful tumor accumulation. We did not observe a major difference in tumor targeting of P-cadherin LP-DART in the presence or absence of immune cells (Supplementary Figure 4A and 4B) suggesting that the tumor targeting was primarily dependent on the tumor antigen. We did not see significant differences between levels of Control LP-DART and P-cadherin LP-DART in any other tissues as the molecules bind specifically to human epitopes. Evaluating the biodistribution using a mouse reactive P-cadherin LP-DART or human CD3 expressing transgenic mouse could provide further insight into this finding. Biodistribution studies with labeled molecules have shown increased distribution of CD3 bispecifics to T cell rich tissues such as the lymph node and spleen in human CD3e transgenic mouse model. Accumulation in normal tissues like liver and spleen may act as a sink and impact the plasma and tumor exposure [25]. Such labeled reagents and tools provide very valuable information on the on- and off-target binding both in pre-clinical models and in patients. It may also provide information on potential target-mediated safety concerns that may be anticipated in patients and help in planning the mitigation strategy [20, 26, 27].

The pharmacology of the bispecific antibody is elicited by forming a bridge between the target (i.e., tumor) cell and the effector (T cell) cell [28]. In order to demonstrate the mechanism of P-cadherin LP-DART we imaged of both drug and the T cell simultaneously. Advances in immuno-therapy, along with the interest in tracking immune cells *in vivo* in patients and in pre-clinical models, has resulted in the development of multiple molecular imaging methodologies [29]. Among them optical imaging, magnetic resonance imaging (MRI) and PET have emerged as major players. In this study we took advantage of the multi-channel capability of FMT imaging and narrow fluorescence spectrums of near-infrared fluorophores which result in minimum contribution across channels [18, 20]. We labeled the T cells with CV815, a lipophilic dye which has a polar head group and an aliphatic hydrocarbon chain. The aliphatic chain gets non-covalently incorporated into the lipid bilayer of the cells. To our knowledge this is the first study to demonstarte non-invasively the dynamics and simultaneous trafficking of T cells and a bispecific antibody to tumors. Similar to our previous observation by immunohistochemistry [16], we saw significantly higher number of T cells in the tumors (Figure 6C). Although CV815 based T cell tracking has several advantages, it may underestimate the number of T cells since the fluorescence is diluted by one-half at each subsequent division of the proliferating T cell. Additionally, since optical imaging has not yet been adopted as a non-invasive modality in clinic, its application is limited to mechanistic studies in pre-clinical models. It will be interesting to evaluate the trafficking of T cells using newer, potentially translational, PET imaging based immuno-oncology biomarkers like IL-2, Granzyme B and CD8-minibody [30–33]. In summary, using FMT imaging we have shown the targeting of P-cadherin LP-DART (Pillar 1 and 2) and the trafficking of T cells (Pillar 3) to the tumor microenvironment in pre-clinical models. P-cadherin LP-DART (PF-06671008) is currently in Phase 1 trials evaluating safety, pharmacokinetics and pharmacodynamics of increasing doses of PF-06671008 in patients with advanced solid tumors. This study has great translational relevance and can aid as a proof of principle for the use of molecular imaging in patients to assess three pillars.

## MATERIALS AND METHODS

### Cell culture

HCT116 (ATCC^®^ CCL-247™) cells line were purchased from American Type Culture Collection (ATCC) and cultured as per their instructions. Cell lines were authenticated, and pathogen tested by IDEXX Bioresearch prior to *in vivo* studies.

### Bispecific antibodies and fluorophore labeling

The bispecific molecules used in this study were produced at Pfizer Inc. The bispecific antibodies P-cadherin DART, a single chain diabody (scDb) (mol. wt. of ~50 kDa), P-cadherin LP-DART (with mol. wt. of ~100 kDa) (Figure 1A–1D) and Control LP-DART (with mol. wt. of ~100 kDa) were labeled with VT680 using a VivoTag^®^680XL protein labeling kit as per the manufacturerʹs instructions (Perkin Elmer Inc) and has been previously described [17]. Briefly, the VT680 has a succinimidyl ester group, which reacts with the primary amine group on the bispecific antibodies to form a stable amide linkage. The reactions were carried out in PBS and after conjugation reaction the free fluorophore was removed using a protein purification column. Degree of labeling (DOL) of VT680 conjugation to bispecific antibodies was evaluated by measuring the absorbance at 280 nm and 668 nm using a NanoDrop™ 8000 (Thermo Fisher Scientific). Reagents with DOL between 1–3 VT680 molecules/protein were used in this study.

### Isolation PBMNCs and expansion of human CD3+ lymphocytes

Whole blood in EDTA was freshly isolated from healthy human donors who were provided with informed consent. Peripheral blood mononuclear cells (PBMNCs) were isolated by density gradient centrifugation layered over Histopaque^®^ 1077 in ACCUSPIN™ conical tubes (Sigma-Aldrich) following the manufacturer’s protocol. PBMNCs were washed twice with Dulbecco’s phosphate buffered saline (DPBS) containing 2 mM EDTA and subjected to further fractionation by negative magnetic bead selection to isolate CD3+ lymphocytes using a human CD3+ negative isolation kit (Stem Cell Technologies). CD3+ lymphocytes were resuspended with CTS™ OpTmizer™ T-Cell Expansion Serum-Free Media supplemented with 2× GlutaMax-1, 1% PenStrep and 20 ng/mL recombinant human IL-2 (Thermo Fisher Scientific). Dynabeads™ Human T-Expander CD3/CD28 magnetic beads (Thermo Fisher Scientific) were added to T-cells at a ratio of 2 beads per cell and cultured for one week. At the time of harvest, beads were removed with a magnet and cells were resuspended in DPBS for *in vivo* inoculation or in Recovery™ Cell Culture Freezing Medium (Thermo Fisher Scientific) for cryopreservation.

### T cell labeling with CellVue™ NIR815 fluorophore

T cells were labeled with CellVue^®^ NIR815 fluorophore (here-after referred as CV815) using CellVue^®^ NIR815 Fluorescent Cell Labeling Kit as per the manufacturerʹs instructions (Thermo Fisher Scientific). T cells (1 × 10^7^ cells/mL) were labeled at the final dye concentration of 2 µM in serum free medium for 3 min and the residual dye after reaction was quenched by serum. After labeling the viability of T cells was evaluated and reactions with viability > 95% were used for the *in vitro* functional assays and *in vivo* studies. To evaluate the T cell expansion *in vitro* CV815-T cells were incubated in CTS™ OpTmizer™ T-Cell Expansion Serum-Free Media supplemented with 2× GlutaMax-1, 1% PenStrep and 20 ng/mL recombinant human IL-2. For stimulation the Dynabeads™ Human T-Expander CD3/CD28 magnetic beads were added to T-cells at a ratio of 2 beads per cell and cultured for 48 hr. The number of viable cells were quantified using CellTiter-Glo^®^ Luminescent Cell Viability Assay (Promega Life Sciences). To assess the effect of CV815 labeling on cytotoxic activity of CD3+ T cells firefly luciferase-transfected HCT116 cells (T) that endogenously expressed P-cadherin were mixed with human CD3+ lymphocytes (E) at different E: T ratios (20:1 to 0.312:1) with 20 pM P-cadherin LP-DART. After 24 h of incubation at 37°C under 5% CO_2_ atmosphere the remaining luciferase activity from the viable tumor target cells was assessed by addition of an equal volume ONE-Glo™ luciferase assay reagent (Promega Life Sciences).

### P-cadherin and CD3 binding ELISA

The binding of VT680 labeled bispecific antibodies to soluble P-cadherin and CD3 proteins were evaluated by ELISA. The soluble P-cadherin and CD3 proteins were expressed using Pfizer’s hu-CD3 epsilon-delta his8 and cyno-P-cadherin-his6 constructs as described previously [10]. ELISA plates were coated overnight with 1 µg/mL huCD3 or cyno-P-cadherin soluble proteins followed by incubation of test samples serially diluted in blocking buffer (3% milk in PBS) for 1 hr at room temperature. Plates were washed with PBS and bound proteins were detected using anti-human IgG-HRP diluted in blocking buffer. Plates were washed, and the bound complexes were detected with TMB substrate. Reaction was stopped with H_2_SO_4_ and the absorbance was measured at 450 nm. Each concentration was evaluated in triplicates. EC50 was determined by non-linear curve fit of relative absorbance using GraphPad Prism 7.04 software (GraphPad Software).

### Cytotoxic T lymphocyte (CTL) activity

CTL assay was performed as described before [16]. Briefly, firefly luciferase transfected HCT116 cells (T) that endogenously expressed P-cadherin were mixed with human CD3+ lymphocytes (E) at the ratio of 5:1, E: T. VT680 labeled P-cadherin LP-DART, P-cadherin DART (scDb) and Control LP-DART bispecific antibodies were added at serial dilutions and incubated for 24 hr at 37°C under 5% CO_2_ atmosphere. The remaining luciferase activity from the viable tumor target cells after 24 hr was assessed by addition of an equal volume ONE-Glo™ luciferase assay reagent (Promega Life Sciences). Relative light units (RLU) were collected with an Envision plate reader (Perkin Elmer Inc.) as counts per second (cps). Percent cytotoxicity was determined by the following formula, 100–100^*^(RLU value of test samples/RLU of control samples). Each concentration was evaluated in triplicate. EC50 was determined by non-linear curve fit of percent cytotoxicity using GraphPad Prism 7.04 software (GraphPad Software).

### *In vivo* biodistribution study

All animal studies were performed as per the animal use protocols approved by Pfizer Inc’s Institutional Animal Care and Use Committee (IACUC) in an AAALAC accredited facility. Animals were housed under standard 12:12 light: dark cycle in individually ventilated cages at a room temp of 72°F (± 2F), relative humidity between 30-70% and were provided with food and water, *ad libitum*.

Biodistribution of P-cadherin LP-DART was evaluated in a HCT116 subcutaneous xenograft model. HCT116 cells (5 × 10^6^ cells/mouse) mixed with 4 mg/mL Cultrex^®^ basement membrane extract (Trevigen) were implanted s. c. on the dorsal right flank of 6–8-week-old NSG or nu/nu female mice purchased from The Jackson Laboratory or Charles River Laboratories. After implantation tumors were measured twice weekly by calipers. Imaging studies were initiated when the tumors were ~300 mm^3^ in volume. The feed for the mice was changed to irradiated alfalfa free diet (AIN76A from Newco Distributors Inc.) 48–72 hr prior to the start of imaging. The animals were injected i. v or s. c. with probes normalized to 1 nmol VT680 concentration (calculated based on DOL). Non-invasive FMT imaging (whole-body) was performed longitudinally post-injection of the probes at various time points, up to 240 hr. Animals were anesthetized with 2–3% isoflurane and maintained under anesthesia in a 37°C warm chamber during each imaging session. For *ex vivo* imaging time points representative animals from each group were perfused under isoflurane anesthesia with PBS/Saline to remove the blood. After perfusion and confirmation of euthanasia, tissues (tumor, liver, spleen, kidney, brain and lungs) were immediately collected for *ex vivo* FMT imaging. Quantitation of FMT data was done using TrueQuant^®^ software (Perkin Elmer Inc). The fluorescence signal measured at 5 min post-injection was used to calculate the percent injected dose (100%ID). Each imaging group had 3–6 animals for each time point.

### *In vivo* evaluation of T cell trafficking

T cells were labeled with CV815 as mentioned before. A pilot *in vivo* study was performed in naïve 6–8-week old female NSG mice with different concentrations of CV815-T cells (1, 5 and 10 × 10^6^ cells) to determine the dose with optimal signal. To evaluate the trafficking of T cell to tumors upon treatment with P-cadherin LP-DART, we used the human T cell adoptive transfer model bearing HCT116 subcutaneous xenograft model. HCT116 cells were implanted in NSG mice as described above and study was initiated when the tumors were ~300 mm^3^. Animals were treated with P-cadherin LP-DART (Unlabeled or Labeled with VT680, dose –2 mg/kg) s. c. on day 0. CV815-T cells (5 × 10^6^ cells/mice) were injected i. v. 24 hr post-injection of drugs (day 1). Multi-channel non-invasive FMT imaging was performed on day 0 (for P-cadherin LP-DART-VT680), day 1, 2, 3, 5, 8 and 11 (for CV815-T cells and P-cadherin LP-DART-VT680, groups as applicable) post-treatment with P-cadherin LP-DART.

## SUPPLEMENTARY MATERIALS



## Abbreviations

3D: Three-dimensional
Ab: Antibody
ADCC: Antibody-dependent cellular cytotoxicity
CDC: Complement-dependent cytotoxicity
CTL: Cytotoxic T lymphocyte
CV815: CellVue^®^NIR815
DART: Dual-affinity re-targeting
DOL: Degree of labeling
EC50: Half maximal effective concentration
EFN: Effector function null mutations
EpCAM: Epithelial cell adhesion molecule
E:T ratios: Effector to target cell ratios
FcRn: Neonatal Fc receptor
FMT: Fluorescence molecular tomography
ID: Injected dose
MRI: Magnetic resonance imaging
NHS: N-hydroxy succinimidyl ester
NIR: Near-infrared
PBMNC: Peripheral blood mononuclear cell
PET: Positron emission tomography
RLU: Relative light unit
ROI: Region of Interest
scDb: Single-chain diabody
scFv: Single-chain variable fragment
TCR: T cell receptor
TMB: 3,3′,5,5′-tetramethylbenzidine
VT680: VivoTag^®^680XL
