# Supplementary material for: Molecular imaging reveals biodistribution of P-cadherin LP-DART bispecific and trafficking of adoptively transferred T cells in mouse xenograft model
Source: Oncotarget. 2020 Apr 14;11(15):1344–57. doi: 10.18632/oncotarget.27544 (PMC7170501; doi:10.18632/oncotarget.27544)
Supplement: Supplementary file 1 [file oncotarget-11-1344-s001.pdf]

Molecular imaging reveals biodistribution of P-cadherin LP-DART bispecific and trafficking of adoptively transferred T cells in mouse xenograft model

SUPPLEMENTARY MATERIALS

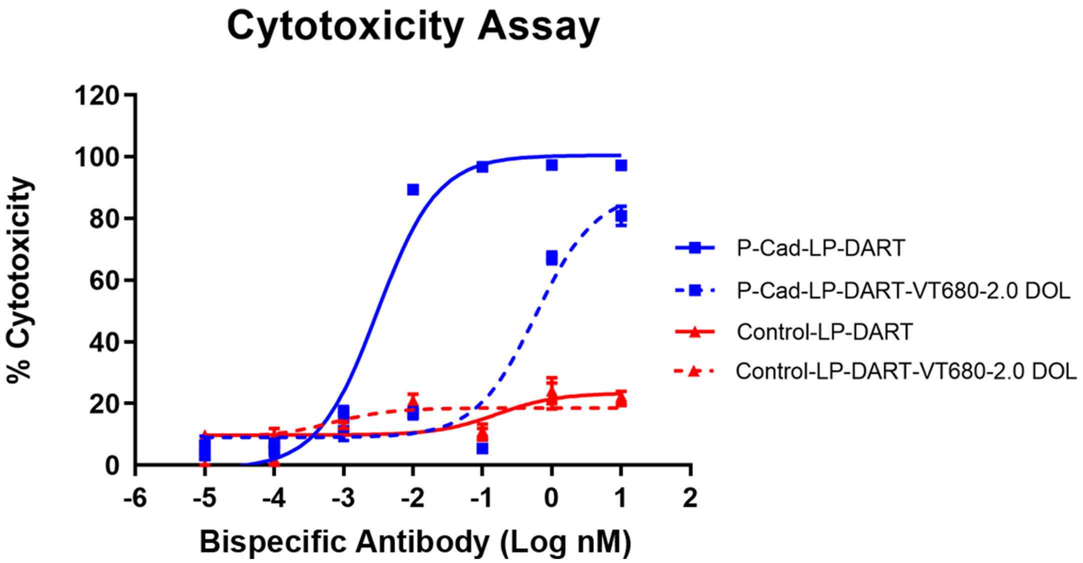

Supplementary Figure 1: CTL assay: Firefly luciferase expressing HCT116 cells and expanded human CD3+ T lymphocytes were co-incubated with VT680 labeled (DOL: 2.0) or unlabeled P-cadherin LP-DART and control LP-DART at different concentrations. After 24 hr the remaining viable cells were quantified by measuring the luciferase activity. The relative cytotoxicity profile suggested that VT680 labeling the P-cadherin LP-DART at DOL of 2.0 reduced the cytotoxic activity. No cytotoxic activity was observed in labeled or unlabeled (DOL 2.0) Control LP-DART at any concentration.

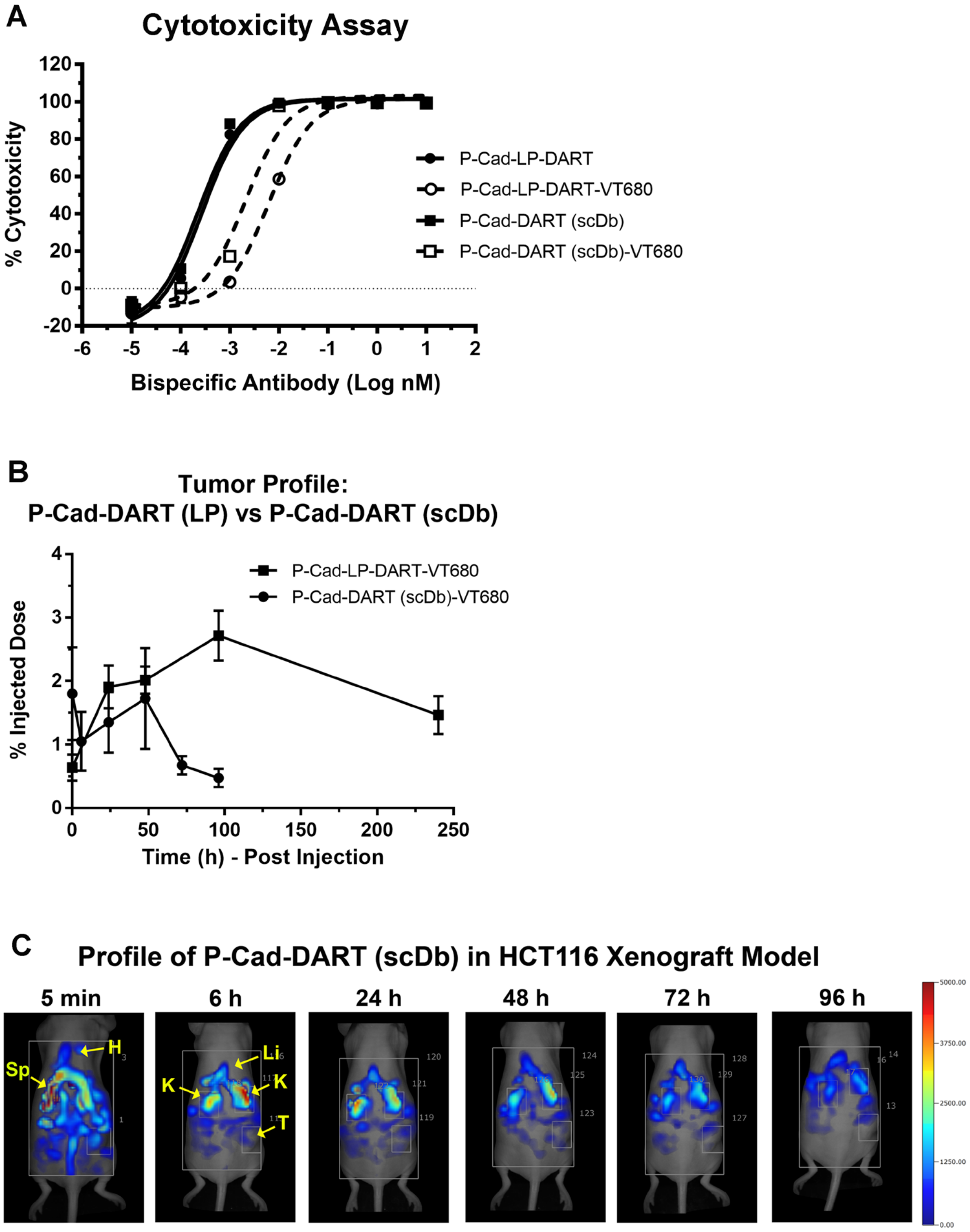

Supplementary Figure 2: Comparison of biodistribution of P-cadherin LP-DART and P-cadherin DART (scDb). P-cadherin DART clears very rapidly from the system, thus limiting tumor exposure compared to P-cadherin LP-DART. (**A**) Cytotoxic activity of VT680 labeled and unlabeled P-cadherin DART and P-cadherin LP-DART. VT680 labeling decreased the cytotoxic activity of the bispecific antibodies. (**B**) *In vivo* tumor profile shows rapid clearance of P-cadherin DART compared to P-cadherin LP-DART. (**C**) *In vivo* FMT imaging profile demonstrates the clearance of P-cadherin DART through kidneys and minimal accumulation in kidneys. No significant kidney specific signal was observed for P-cadherin LP-DART (Figure 3A). There was no detectable signal of P-cadherin DART in any tissues other than kidneys at 96 hr upon *ex vivo* quantitation (data not shown).

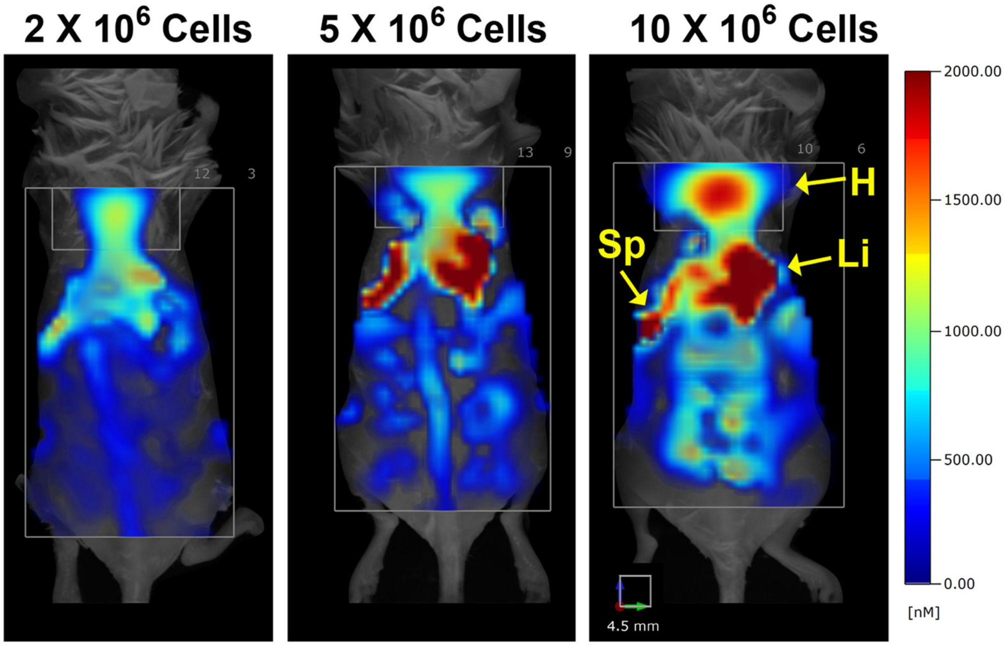

Supplementary Figure 3: FMT images 24 hr post-adoptive transfer showing the whole-body distribution of CV815-T cells in 6–8 week old Naïve NSG mice at different concentrations (2 × 10⁶; 5 × 10⁶; 10 × 10⁶ T cells). Images showed high number of T cells in liver, spleen and circulation (heart and GI signal). This pilot study helped select the cell number (5 × 10⁶ T cells) for the T cell trafficking study.

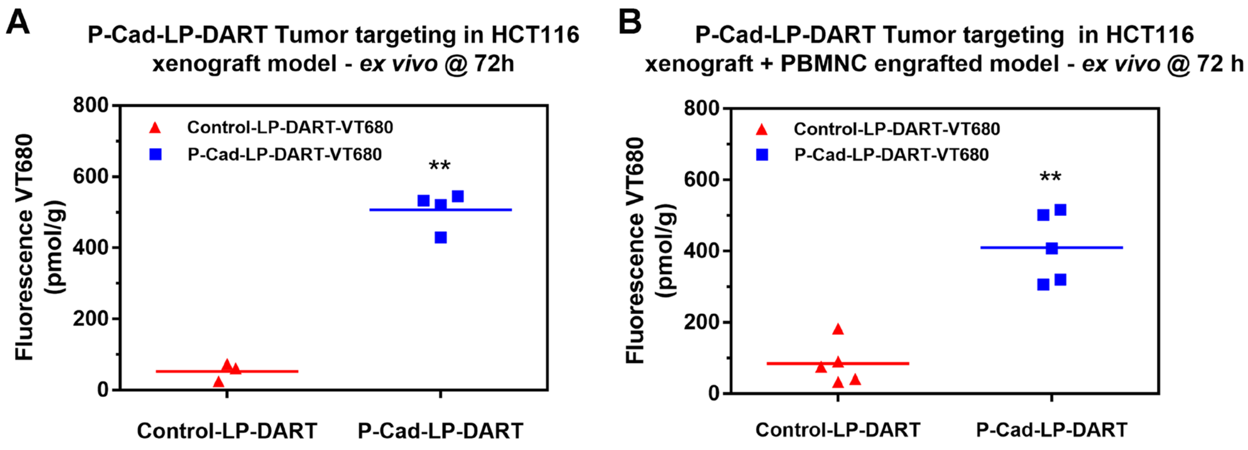

Supplementary Figure 4: *Ex vivo* FMT quantitation shows no major difference in tumor targeting of P-cadherin LP-DART between (**A**) non-engrafted vs (**B**) PBMNC engrafted HCT116 xenografts. $n = 3–5$/group. HCT116 cells (5×10^6 cells/mouse) mixed with 4 mg/mL Cultrex basement membrane extract was implanted s. c. on the dorsal right flank of 6–8-week-old NSG female mice. Seven days before randomization mice were inoculated with freshly isolated 5×10^6 PBMNC via i. p. route. Mice bearing 200–300 mm^3 tumors were enrolled to evaluate the effect of engraftment on P-cadherin LP-DART targeting to tumor.
